# Supplementary material for: SARS-CoV-2 Spike Glycoprotein S1 Induces Neuroinflammation in BV-2 Microglia
Source: Mol Neurobiol. 2021 Oct 28;59(1):445–58. doi: 10.1007/s12035-021-02593-6 (PMC8551352; doi:10.1007/s12035-021-02593-6)

SARS-CoV-2 spike glycoprotein S1 induces neuroinflammation in BV-2 microglia

Olumayokun A Olajide[+*], Victoria U Iwuanyanwu, Oyinkansola D Adegbola, Alaa A Al-Hindawi

Department of Pharmacy, School of Applied Sciences, University of Huddersfield, Queensgate, Huddersfield, HD1 3DH, United Kingdom

[+] ORCID ID: 0000-0002-9254-8334

***Address for correspondence:**

Dr Olumayokun A Olajide
Department of Pharmacy, University of Huddersfield
Queensgate, Huddersfield, HD1 3DH, United Kingdom
Email: o.a.olajide@hud.ac.uk

Supplementary Data

Effects of 10, 50, 100, 500 and 1000 ng/mL of S1 on TNFα production following stimulation of BV-2 microglia for 1, 3, 6, 12 and 24 h

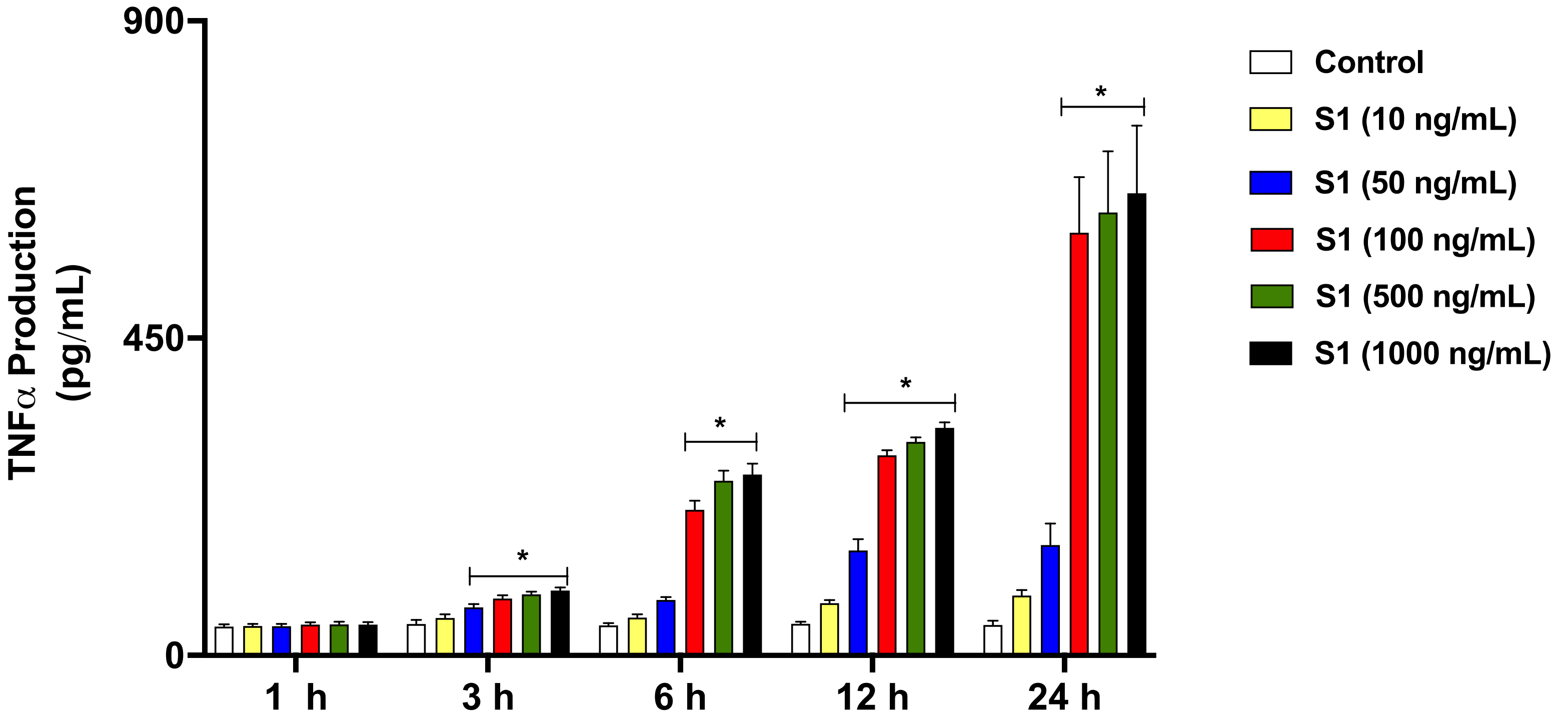

Supplement: Supplementary file 1 — Supplementary file1 (PDF 43 KB) [file 12035_2021_2593_MOESM1_ESM.pdf]
